# Snoring and Glaucoma

**DOI:** 10.1371/journal.pone.0088949

**Published:** 2014-02-13

**Authors:** Ya Xing Wang, Liang Xu, Jian Jun Li, Hua Yang, Ya Qin Zhang, Jost B. Jonas

**Affiliations:** 1 Beijing Institute of Ophthalmology, Beijing Tongren Hospital, Capital University of Medical Science, Beijing, China; 2 Department of Ophthalmology, Faculty of Clinical Medicine Mannheim, University of Heidelberg, Mannheim, Germany; Massachusetts Eye & Ear Infirmary, Harvard Medical School, United States of America

## Abstract

**Purpose:**

To examine a potential association between snoring and glaucoma in a population-based setting.

**Methods:**

The population-based Beijing Eye Study 2011 included 3468 subjects with an age of 50+ years. The participants underwent a detailed ophthalmic examination. Glaucoma was determined according to the ophthalmoscopic appearance of the optic nerve head. Snoring assessed in an interview was graded into “severe snoring”, “moderate snoring”, and “no snoring”.

**Results:**

Data on snoring and glaucoma were available for 3146 subjects. Snoring was reported for 1787 (66.8%) subjects, with moderate snoring reported for 1384 (44.0%) subjects and severe snoring for 403 (12.8%) subjects. In multivariate analysis, prevalence of severe snoring was significantly associated with male gender (P = 0.002; regression coefficient B: 0.36; Odds ratio (OR): 1.44 (95% confidence interval (CI): 1.14, 1.81)), higher body mass index (*P*<0.001; B: 0.12; OR: 1.13 (95%CI: 1.09, 1.16)), higher systolic blood pressure (*P*<0.001; B: 0.01; OR: 1.01 (95%CI: 1.005, 1.02)), younger age (*P* = 0.007; B: −0.018; OR: 0.98 (95%CI: 0.97, 0.995)), and higher cognitive function (*P* = 0.03; B: 0.04; OR: 1.04 (95%CI: 1.004, 1.08)), however it was not significantly associated with the prevalence of open-angle glaucoma (*P* = 0.10; B: −0.63; OR: 0.53 (95%CI: 0.25, 1.12)). Prevalence of severe snoring was neither significantly associated with the prevalence of angle-closure glaucoma (*P* = 0.65), retinal vein occlusions (*P* = 0.24), neuroretinal rim area (*P* = 0.19), retinal nerve fiber layer thickness (*P* = 0.16) nor vertical cup/disc ratio (*P* = 0.64).

**Conclusions:**

Severe snoring was not significantly associated with the prevalence of open-angle glaucoma, angle-closure glaucoma or retinal vein occlusions after adjustment for age, gender, body mass index, systolic blood pressure and cognitive function score. Our population-based study did not reveal that snoring was a risk factor for glaucoma and thus did not provide a reason to assess or to treat snoring in patients with glaucoma.

## Introduction

Sleep apnea syndrome (SAS) is a disorder characterized by recurrent complete or partial obstructions of the upper airways during sleep [1]. Besides choking, snorting or gasping during sleep, loud and chronic snoring is one of the major signs and symptoms of sleep apnea [1]. Severe hypoxia and hypercapnia can develop during the obstructive respiratory disturbances which can last up to 2 minutes. Several hundreds of respiratory disturbances can occur during one night and can cause severe sleep disruption with consequential daytime sleepiness. Middle-aged or elderly, obese men with a long lasting history of loud snoring are usually affected. After Hayreh discussed a potential association between sleep apnea and glaucomatous optic neuropathy, several studies have examined whether sleep apnea is associated with, or even a risk factor for, chronic open-angle glaucoma or other vascular optic neuropathies such as anterior ischemic optic neuropathy [2]–[7]. These hospital-based studies had as major limiting factor a potential selection bias due to the referral of the patients and were not conclusive in their results. While studies by Mojon and colleagues reported a clear association between sleep apnea and glaucoma, Geyer and coworkers did not find a significant difference in the prevalence of glaucoma between patients with sleep apnea and the general population [3], [4]. In a recent large-scaled retrospective, matched-cohort study by Li and colleagues [8], the hazard ratio for the development of open-angle glaucoma during a 5-year follow-up period was significantly elevated for subjects with obstructive sleep apnea, after adjusting for parameters such as monthly income, geographic region and presence of systemic diseases. That study however had the limitations of its retrospective study design and the use of data of an insurance database with no direct control of the diagnoses.

Having the potential referral bias as major limiting factor in the previous investigations in mind and taking a subjectively assessed degree of snoring as surrogate for the presence of sleep apnea, we performed this population-based study to evaluate and compare the prevalence of different degrees of snoring in a general population divided into patients with glaucoma and into non-glaucomatous subjects. Although taking into account that the diagnostic criteria employed to subjectively assess snoring in such a study may be a limiting factor, the knowledge of a relationship between sleep apnea and glaucoma may be of clinical importance since if it exists, preventive measures such as sleep apnea therapy could be advised to reduce the risk of glaucoma or glaucoma progression.

## Methods

### Ethics Statement

The Medical Ethics Committee of the Beijing Tongren Hospital approved the study protocol and all participants gave informed written consent, according to the Declaration of Helsinki.

The Beijing Eye Study 2011 is a population-based study in Northern China [9], [10]. It was carried out in 5 communities in the urban district of Haidian in the North of Central Beijing and in 3 communities in the village area of Yufa of the Daxing District south of Beijing. The only eligibility criterion for inclusion into the study was an age of 50+ years. In 2011, the 8 communities had a total population of 4403 individuals aged 50 years or older. Out of the 4403 eligible subjects, 3468 individuals (1963 (56.6%) women) participated in the eye examination, corresponding to an overall response rate of 78.8%. The study was divided into a rural part (1633 (47.1%) subjects; 943 (57.7%) women) and an urban part (1835 (52.9%) subjects; 1020 (55.6%) women). The mean age was 64.6±9.8 years (median, 64 years; range, 50–93 years). The study was described in detail recently. All study participants underwent an interview with standardized questions on their family status, level of education, income, quality of life, psychic depression, physical activity, known major systemic diseases such as arterial hypertension and diabetes mellitus and quality of vision. Snoring was assessed by asking the life partner and was graded into “no snoring or only mild snoring”; “moderate snoring”; and “severe snoring”. If no information could be obtained, the subject was not included into the present investigation. Cognitive function was assessed using the MMSE (mini mental state examination) scale [11]. Fasting blood samples were taken for measurement of blood lipids, glucose and glycosylated hemoglobin HbA1c. Blood pressure was measured. Body height and weight and the circumference of the waist and hip were recorded. The ophthalmic examination included measurement of presenting visual acuity and uncorrected visual acuity. Refractive error was assessed by automatic refractometry (Auto Refractometer AR-610, Nidek Co., Ltd, Tokyo, Japan), if uncorrected visual acuity was lower than 1.0. Best corrected visual acuity was assessed by subjective refractometry. Intraocular pressure was measured by pneumotonometry by an experienced ophthalmologist. A slit lamp examination carried out by an ophthalmologist addressed lid abnormalities, Meibomian gland dysfunction, corneal disorders, and peripheral anterior chamber depth using van Herick’s method. Using optical low-coherence reflectometry (Lensstar 900® Optical Biometer, Haag-Streit, 3098 Koeniz, Switzerland), biometry of the right eyes was performed for measurement of the anterior corneal curvature, central corneal thickness, anterior chamber depth, lens thickness and axial length. The pupil was dilated using tropicamide once or twice, until the pupil diameter was at least 6 mm. A second slit lamp assisted biomicroscopy searched for pseudoexfoliation syndrome. Digital photographs of the cornea and lens and retro-illuminated photographs of the lens were taken using the Neitz CT-R camera (Neitz Instruments Co., Tokyo, Japan). Monoscopic photographs of the macula and optic disc were taken using a fundus camera (Type CR6-45NM, Canon Inc. U.S.A.). The optic disc size was morphometrically analyzed by spectral domain optical coherence tomography spectral domain OCT (iTVue SD-OCT; Optovue Inc. Fremont, CA, U.S.A.) for measurement of optic disc size, neuroretinal rim area and horizontal and vertical cup/disc ratios. The retinal nerve fiber layer thickness was also measured by spectral domain optical coherence tomography (Spectralis HRA+OCT, Heidelberg Engineering, Heidelberg, Germany).

Glaucoma was defined in two manners. First, glaucoma was defined according to the optic nerve head criteria of the International Society of Geographic and Epidemiological Ophthalmology ISGEO [12]. Second, examining the digital photographs of the optic disc and macula, glaucoma was defined by a glaucomatous appearance of the optic disc. The optic nerve head was glaucomatous (1) if the inferior-superior-nasal-temporal (ISNT)-rule of the neuroretinal rim shape was not fulfilled in early glaucoma and in eyes with a normally shaped optic disc (it included a notch in the neuroretinal rim in the temporal inferior region and/or the temporal superior region); or (2) if an abnormally large cup was present in a small optic disc which normally would not show cupping. The assessment of the optic disc photographs was carried out in a masked manner. Each photograph of a glaucomatous optic disc was independently adjudicated by three senior graders (LX, YXW, JBJ). As described in detail previously [13], the whole glaucoma group was then differentiated by gonioscopy or anterior segment optical coherence tomography into subjects with open-angle glaucoma or subjects with angle-closure glaucoma.

Only those subjects with available data on snoring and on glaucoma were included into the study described herein. Statistical analysis was performed using a commercially available statistical software package (SPSS for Windows, version 20.0, IBM-SPSS, Chicago, IL). In a first step, we determined the mean values (presented as mean ± standard error for frequencies; as mean ± standard deviation for all other parameters) and medians of the main outcome parameters. In a second step, we performed univariate analyses of the associations between the prevalence of severe snoring and systemic and ocular other parameters including the prevalence of glaucoma. In a third step, we carried out a binary regression analysis with the prevalence of severe snoring as dependent parameter and all those variables as independent parameters for which the *P*-value was ≤0.10 in the univariate analysis of their associations with severe snoring. In a fourth step, we removed from the multivariate analysis, step-by-step, those parameters that were no longer associated significantly with severe snoring, starting with those parameters that showed the least probable association (highest P-value). Odds ratios (OR) and 95% confidence intervals (CI) were presented.

## Results

Data on snoring and the presence of glaucoma were available for 3146 subjects with a mean age of 64.4±9.7 years (median, 63 years; range, 50–93 years), and the mean refractive error was –0.20±2.10 diopters (median; +0.25 diopters; range; −22.00 diopters to +7.00 diopters). Overall prevalence of glaucoma was 5.94±0.53% (95%CI: 4.91, 6.98); prevalence of open-angle glaucoma was 2.96±0.30 (95%CI: 2.36, 3.55), and of primary angle-closure glaucoma 1.40±0.21% (95%CI: 0.99, 1.81), and secondary angle closure glaucoma 0.06±0.05% (95%CI: 0.00, 0.15). Snoring was graded as “mild or not existent” for 1359 (43.2%) subjects, as “moderate” for 1384 (44.0%) subjects, and as “severe” for 403 (12.8%) subjects.

In univariate analysis, severe snoring was significantly associated with the systemic parameters of male gender (*P* = 0.004), taller body height (*P* = 0.006), higher body weight (*P*<0.001), higher body mass index (*P*<0.001), younger age (*P*<0.001), rural region of habitation (*P*<0.001), ever smoking (*P*<0.001), package years of smoking (*P*<0.001), cognitive level (*P* = 0.03), higher systolic and diastolic blood pressure (*P*<0.001), and the ocular parameters of higher prevalence of open-angle glaucoma (*P* = 0.04), and thicker subfoveal choroidal thickness (*P* = 0.02) (Table 1). The associations between the prevalence of severe snoring and higher intraocular pressure (*P* = 0.06) and a lower the prevalence of retinal vein occlusions (*P* = 0.06) were statistically marginal. Prevalence of severe snoring was not significantly (all *P*>0.10) associated with the systemic parameters of level of education, blood concentrations of glucose, low-density lipoproteins, high-density lipoproteins, cholesterol and triglycerides, drinking alcohol, and with the ocular parameters of refractive error, axial length, best corrected visual acuity, optic disc size, neuroretinal rim area, horizontal and vertical cup/disc ratio, retinal nerve fiber layer thickness, and the prevalence of angle-closure glaucoma, early age-related macular degeneration, and diabetic retinopathy (Table 1).

**Table 1:** Associations (univariate analysis) between the prevalence of severe snoring and ocular and systemic parameters in the Beijing Eye Study 2011.

Parameter	*P*-Value		RegressionCoefficient B			Odds Ratio	95% Confidence Interval
Systemic Parameters							
Age (Years)		<0.001		−0.025	0.98		0.96, 0.99
Women/Men		0.004			1.36		1.10, 1.68
Body Height (cm)		0.006		0.018	1.02		1.01, 1.03
Body Weight (kg)		<0.001		0.05	1.05		1.04, 1.06
Body Mass Index (kg/m^2^)		<0.001		0.13	1.14		1.11, 1.17
Rural/Urban Region of Habitation		<0.001		0.63	0.63		0.51, 0.77
Level of Education		0.36					
Cognitive Level		0.03		0.04	1.04		1.00, 1.07
Smoking Ever		<0.001			1.50		1.21, 1.87
Package Years of Smoking		<0.001		0.10	1.10		1.05, 1.16
Drinking Alcohol		0.21					
Blood Glucose Concentration (mmol/L)		0.12		0.05	1.05		0.99, 1.13
Glycosylated Hemoglobin HbA1c		0.08		0.09	1.09		0.99, 1.22
Blood Concentration of Low-Density Lipoproteins (mmol/L)		0.13		0.10	1.10		0.97, 1.26
Blood Concentration of High-Density Lipoproteins (mmol/L)		0.25					
Blood Concentration of Cholesterol (mmol/L)		0.95					
Blood Concentration of Triglycerides (mmol/L)		0.52					
Blood Concentration of Creatinine (µmol/l)		0.72					
Systolic Blood Pressure (mmHg)		<0.001		0.012	1.01		1.01, 1.02
Diastolic Blood Pressure (mmHg)		<0.001		0.025	1.03		1.02, 1.03
Mean Blood Pressure (mmHg)		<0.001		0.02	1.02		1.01, 1.03
Ocular Parameters							
Refractive Error (Diopters)		0.34					
Axial Length (mm)		0.13		−0.08	0.93		0.84, 1.02
Best Corrected Visual acuity (logMAR)		0.56					
Intraocular Pressure (mmHg)		0.06		0.04	1.04		1.00, 1.08
Subfoveal Choroidal Thickness (µm)		0.02		0.001	1.001		1.000, 1.001
Retinal Nerve Fiber Layer Thickness (µm)		0.16					
Optic Disc Area (mm^2^)		0.56					
Neuroretinal Rim Area (mm^2^)		0.19					
Vertical Cup/Disc Ratio		0.64					
Horizontal Cup/Disc Ratio		0.81					
Presence of Glaucoma		0.08		−0.47	0.63		0.37, 1.06
Open-Angle Glaucoma		0.04		−0.75	0.47		0.23, 0.97
Angle-Closure Glaucoma		0.65		−0.19	0.83		0.38, 1.84
Diabetic Retinopathy		0.34					
Early Age-Related Macular Degeneration		0.18			1.42		0.88, 2.27
Presence of Retinal Vein Occlusion		0.06			0.27		0.07, 1.11

In multivariate analysis, we included the prevalence of severe snoring as dependent parameter and all those variables as independent parameters for which the P-value was ≤0.10 in the univariate analysis. We then removed from the list of independent parameters step-by-step those parameters that were no longer associated significantly with severe snoring, starting with the parameters with the highest P-value. After stepwise removal of diastolic blood pressure (*P* = 0.97), intraocular pressure (*P* = 0.81), body height (*P* = 0.73), region of habitation (*P* = 0.59), ever smoking (*P* = 0.52), percentage of glycosylated hemoglobin HbA1c (*P* = 0.48), subfoveal choroidal thickness (*P* = 0.19) and prevalence of retinal vein occlusions (*P* = 0.055), the prevalence of severe snoring was significantly associated with younger age (*P* = 0.007), male gender (*P* = 0.002), higher body mass index (*P*<0.001), higher systolic blood pressure (*P*<0.001) and higher cognitive function (*P* = 0.03), however it was not significantly associated with the prevalence of open-angle glaucoma (*P* = 0.10; regression coefficient B: −0.63; OR: 0.47 (95%CI: 0.25, 1.12)) (Table 2). The same result was obtained if the ISGEO definition of glaucoma was applied: the prevalence of severe snoring was associated with younger age (*P* = 0.03), male gender (*P* = 0.003), higher body mass index (*P*<0.001), higher systolic blood pressure (*P*<0.001) and higher cognitive function (*P* = 0.02), however it was not significantly associated with the prevalence of glaucoma (*P* = 0.29; regression coefficient B: −0.50; OR: 0.60 (95%CI: 0.24, 1.54)). If the presence of glaucoma was replaced by the area of the neuroretinal rim in the list of independent parameters, severe snoring was no longer significantly associated with neuroretinal rim area (*P* = 0.23; regression coefficient B: 0.25; OR: 1.28 (95%CI: 0.85, 1.93) after adjusting for age, gender, body mass index, systolic blood pressure and cognitive function. If instead of the prevalence of open-angle glaucoma the prevalence of retinal vein occlusions was added to the list of independent parameters, the results were similar in the sense that the prevalence of retinal vein occlusions was not significantly associated with the presence of severe snoring (*P* = 0.24; B: −0.71; OR: 0.49 (95%CI: 0.15, 1.62)).

**Table 2:** Associations (multivariate analysis) between the prevalence of severe snoring and ocular and systemic parameters in the Beijing Eye Study 2011.

Parameter	*P*-Value	RegressionCoefficient B	Odds Ratio	95% Confidence Interval of Odds Ratios
Age (Years)	0.007	−0.018	0.98	0.97, 0.995
Women/Men	0.002	0.36	1.44	1.14, 1.81
Body Mass Index (kg/m^2^)	<0.001	0.12	1.13	1.09, 1.16
Systolic Blood Pressure (mmHg)	<0.001	0.01	1.01	1.005, 1.02
Cognitive Function Score	0.03	0.04	1.04	1.004, 1.08
Prevalence of Open-Angle Glaucoma	0.10	−0.63	0.53	0.25, 1.12

If moderate and severe snoring was included into a “snoring” group, similar results were obtained. In multivariate analysis, after stepwise removal of diastolic blood pressure (*P* = 0.81), ever smoking (*P* = 0.83), subfoveal choroidal thickness (*P* = 0.54), percentage of glycosylated hemoglobin HbA1c (*P* = 0.41), intraocular pressure (*P* = 0.89), body height (*P* = 0.12), and systolic blood pressure (*P* = 0.27), the prevalence of moderate combined with severe snoring was significantly associated with younger age (*P* = 0.02; B: −0.01; OR: 0.99; 95%CI: 0.98, 0.999), male gender (*P* = 0.010; B: 0.24; OR: 1.27; 95%CI: 1.06, 1.52), higher body mass index (*P*<0.001; B: 0.13; OR: 1.13; 95%CI: 1.11, 1.16), higher cognitive function (*P* = 0.004; B: 0.03; OR: 1.04; 95%CI: 1.01, 1.06), smoking quantity (*P* = 0.001; B: 0.15; OR: 1.16; 95%CI: 1.06, 1.27), urban region of habitation (*P*<0.001; B: 0.32; OR: 1.37; 95%CI: 1.15, 1.64), and higher prevalence of retinal vein occlusions (*P* = 0.02; B: 0.78; OR: 2.18; 95%CI: 1.11, 4.28), while it was not significantly associated with the prevalence of open-angle glaucoma (*P* = 0.39; B: −0.18; OR: 0.84; 95%CI: 0.56, 1.25).

## Discussion

In our population-based study, severe snoring was significantly associated with younger age (*P* = 0.007), male gender (*P* = 0.002), higher body mass index (*P*<0.001), higher systolic blood pressure (*P*<0.001) and higher cognitive function (*P* = 0.03), while it was not significantly associated with the prevalence of open-angle glaucoma (*P* = 0.10), angle-closure glaucoma (*P* = 0.65) and retinal vein occlusions (*P* = 0.24) nor with neuroretinal rim area (*P* = 0.19), retinal nerve fiber layer thickness (*P* = 0.16) and vertical cup/disc ratio (*P* = 0.64). These findings suggest that severe snoring is not associated with glaucoma.

Our results agree with the hospital-based study by Geyer and colleagues who examined 228 patients with sleep apnea syndrome and found that the prevalence of open-angle glaucoma of 2% (95%CI: 0.7%–5%) in the sleep apnea group was similar to that in the general Caucasian population [5]. In agreement with this finding, Geyer et al. did not detect a significant correlation between the respiratory disturbance index during night sleep as measure for the sleep apnea and the presence of glaucoma (*P* = 0.60) nor with intraocular pressure (*P* = 0.32). Geyer’s and our results are in contrast to recent large-scaled retrospective, matched-cohort study by Li and colleagues [8], Li and coworkers used the Taiwanese Longitudinal Health Insurance Database 2000 as a nationwide, population-based dataset and examined the prevalence and risk of open-angle glaucoma among patients with obstructive sleep apnea during a 5-year follow-up period after a diagnosis of obstructive sleep apnea. They included 1012 subjects with obstructive sleep apnea in the study cohort and randomly selected 6072 subjects in the comparison group. During the 5-year follow-up period, the hazard ratio for open-angle glaucoma for subjects with obstructive sleep apnea was 1.7 (95% CI: 1.3−2.2; *P*<0.001) that of the comparison subjects, after adjusting for monthly income, geographic region, presence of diabetes mellitus, arterial hypertension, coronary heart disease, obesity, hyperlipidemia, renal disease, hypothyroidism, and number of outpatient visits for ophthalmologic care during the follow-up period. Li et al. concluded that obstructive sleep apnea was associated with an increased risk of subsequent open-angle glaucoma diagnosis during a 5-year follow-up period. The results of our study also disagree with the findings from a hospital-based investigation by Mojon and colleagues [3]. Mojon et al. reported that the prevalence of glaucoma in 69 patients with sleep apnea syndrome (5 out of 69 or 7%) was significantly higher than expected in a corresponding population (2%) (*P* = 0.01). In addition, the respiratory disturbance index during night sleep correlated positively with intraocular pressure (*P* = 0.025), visual field loss variance (*P* = 0.03), glaucomatous optic disc changes (*P* = 0.001) and diagnosis of glaucoma (*P* = 0.01). In another smaller-scaled study, Mojon and coworkers examined 8 patients with suspected sleep apnea syndrome [4]. All three patients diagnosed with severe sleep apnea syndrome and one patient with moderate sleep apnea syndrome had relative nasal arcuate visual field defects, and two patients with severe sleep apnea syndrome also had paracentral relative defects. One patient with normal polysomnographic result and two patients with mild or moderate sleep apnea syndrome had normal visual fields. The respiratory disturbance index correlated positively with the perimetric indices (*P*<0.05). Interestingly, in two of the three patients with severe sleep apnea syndrome and treated with continuous positive airway pressure, the visual field defects remained stable over 18 months. Another patient with optic nerve drusen and severe sleep apnea syndrome showed a marked improvement of constricted visual fields after treatment with continuous positive airway pressure. It was also Mojon and his team who found a high prevalence of sleep apnea syndrome in 17 patients with non-arteritic anterior ischemic optic neuropathy [6]. The authors concluded the observed association could explain why the majority of patients with non-arteritic anterior ischemic optic neuropathy discovered the visual loss on first awakening. Behbehani et al. however, reported on 3 out 108 patients with sleep apnea syndrome who developed non-arteritic anterior ischemic optic neuropathy under therapy with continuous positive airway pressure [7]. Finally, in the recent large EPIC-Norfolk eye study by Yip and colleagues on 5650 participants aged 48 to 90 years, lower levels of physical activity were associated with lower ocular perfusion pressure [14]. A low ocular perfusion pressure has been shown to be a risk for glaucomatous optic neuropathy. Since snoring and sleep apnea are associated with a high body mass index which is itself is correlated with a low degree of physical activity, one may infer that the results of the EPIC-Norfolk eye study may favor an association between snoring/sleep apnea and glaucoma. Other studies reported that snoring patients showed a higher prevalence of glaucoma or a familial aggregation of glaucoma in snoring subjects, a reduced retinal nerve fiber layer thickness and an abnormal blood flow [15]–[20]. All these studies were limited by their designs as hospital-based investigations, a low number of patients and the potential effect of confounding parameters.

Summarizing the available information, one may conclude that previous studies, each with limitations such as a relatively small number of patients, missing of matched control groups and hospital-based study designs revealed conflicting results on the relationship between snoring and glaucoma. In the multivariate analysis of our study, severe snoring was by trend associated even with a lower prevalence of glaucoma as indicated by the negative regression coefficients and the odds ratio <1.0 (Table 1,2). This finding was independent of the type of glaucoma definition applied. Fitting with the notion, that glaucoma was not related with severe snoring were the observations that severe snoring was significantly associated neither with neuroretinal rim area, retinal nerve fiber layer thickness or vertical cup/disc ratio, all parameters which serve as surrogates for glaucomatous optic neuropathy, independently of any glaucoma definition. Interestingly, an association between increased cranial pressure and severe snoring/sleep apnea was reported previously [21]. It would point towards a protective role of severe snoring against glaucoma, since a low intracranial pressure has recently been discussed to be a risk factor for glaucoma [22]. The observed associations between severe snoring and male gender, higher body mass index and higher systolic blood pressure are in full agreement with previous general investigations in snoring and sleep apnea [23].

One may argue whether snoring can be used as a surrogate to sleep pathology including sleep apnea. Durán and colleagues examined the prevalence and related clinical features of obstructive sleep apnea-hypopnea in a population-based study in two phases [24]. In a phase including 2148 subjects were examined in a home survey with measurements of blood pressure and respiratory recording. In the second phase, people with suspected obstructive sleep apnea–hypopnea and normal subjects underwent polysomnography. It revealed that habitual snoring as crude data and adjusted for age and gender were strongly (*P*<0.001) associated with the apnea–hypopnea index. It may suggest that snoring may be taken as surrogate for obstructive sleep apnea-hypopnea problems. The assumption to consider snoring as a certain surrogate for obstructive sleep apnea-hypopnea problems has also been discussed by Malhotra and White [25]. Durán and associates reported that habitual snoring was found in 35% of their population, a figure similar to our findings (moderate snoring in 44% of the subjects, severe snoring in 13% of the study participants) [25]. In Durán’s study as well as in our investigation, snoring occurred more frequently in men and increased with age.

Potential limitations of our study should be mentioned. First, as for any prevalence study nonparticipation or exclusion of subjects may have led to a confounding effect. Second, presence and amount of snoring was assessed by asking the life partner. This methodology was clearly inferior to the established method of measuring the respiratory disturbance index in a nocturnal sleep laboratory. It would have been however impossible admitting randomly selected subjects in a high number to a sleep laboratory. The data on snoring in our study may be strengthened by the finding that the prevalence of severe snoring in our study was associated with similar systemic parameters (such as male gender, high body mass index and arterial hypertension) as it was in other studies quantifying snoring and sleep apnea by measuring the respiratory disturbance index.

In conclusion, severe snoring was not significantly associated with prevalence of open-angle glaucoma, angle-closure glaucoma or retinal vein occlusions after adjustment for age, gender, body mass index, systolic blood pressure and cognitive function score. Our population-based study did not reveal that snowing was a risk factor for glaucoma and thus did not provide a reason to assess or to treat snoring in patients with glaucoma.
